# HRPK-1, a conserved KH-domain protein, modulates microRNA activity during *Caenorhabditis elegans* development

**DOI:** 10.1371/journal.pgen.1008067

**Published:** 2019-10-04

**Authors:** Li Li, Isana Veksler-Lublinsky, Anna Zinovyeva

**Affiliations:** 1 Division of Biology, Kansas State University, Manhattan, Kansas, United States of America; 2 Department of Software and Information Systems Engineering, Ben-Gurion University of the Negev, Beer-sheva, Israel; University of Cambridge, UNITED KINGDOM

## Abstract

microRNAs (miRNAs) are potent regulators of gene expression that function in diverse developmental and physiological processes. Argonaute proteins loaded with miRNAs form the miRNA Induced Silencing Complexes (miRISCs) that repress gene expression at the post-transcriptional level. miRISCs target genes through partial sequence complementarity between the miRNA and the target mRNA’s 3’ UTR. In addition to being targeted by miRNAs, these mRNAs are also extensively regulated by RNA-binding proteins (RBPs) through RNA processing, transport, stability, and translation regulation. While the degree to which RBPs and miRISCs interact to regulate gene expression is likely extensive, we have only begun to unravel the mechanisms of this functional cooperation. An RNAi-based screen of putative ALG-1 Argonaute interactors has identified a role for a conserved RNA binding protein, HRPK-1, in modulating miRNA activity during *C*. *elegans* development. Here, we report the physical and genetic interaction between HRPK-1 and ALG-1/miRNAs. Specifically, we report the genetic and molecular characterizations of *hrpk-1* and its role in *C*. *elegans* development and miRNA-mediated target repression. We show that loss of *hrpk-1* causes numerous developmental defects and enhances the mutant phenotypes associated with reduction of miRNA activity, including those of *lsy-6*, *mir-35*-family, and *let-7*-family miRNAs. In addition to *hrpk-1* genetic interaction with these miRNA families, *hrpk-1* is required for efficient regulation of *lsy-6* target *cog-1*. We report that *hrpk-1* plays a role in processing of some but not all miRNAs and is not required for ALG-1/AIN-1 miRISC assembly. We suggest that HRPK-1 may functionally interact with miRNAs by both affecting miRNA processing and by enhancing miRNA/miRISC gene regulatory activity and present models for its activity.

## Author summary

microRNAs are small non-coding RNAs that regulate gene expression at the post-transcriptional level. The core microRNA Induced Silencing Complex (miRISC), composed of Argonaute, mature microRNA, and GW182 protein effector, assembles on the target messenger RNA and inhibits translation or leads to messenger RNA degradation. RNA binding proteins interface with miRNA pathways on multiple levels to coordinate gene expression regulation. Here, we report identification and characterization of HRPK-1, a conserved RNA binding protein, as a physical and functional interactor of miRNAs. We confirm the physical interaction between HRPK-1, an hnRNPK homolog, and Argonaute ALG-1. We report characterizations of *hrpk-1* role in development and its functional interactions with multiple miRNA families. We suggest that HRPK-1 promotes miRNA activity on multiple levels in part by contributing to miRNA processing and by coordinating with miRISC at the level of target RNAs. This work contributes to our understanding of how RNA binding proteins and auxiliary miRNA cofactors may interface with miRNA pathways to modulate miRNA gene regulatory activity.

## Introduction

Robust regulation of gene expression is essential for normal development and cellular homeostasis. microRNAs (miRNAs), small non-coding RNAs ~22nt in length, negatively regulate gene expression at the post-transcriptional level. miRNAs can act as developmental switches or can fine tune the expression of the target genes (for review, see [1], [2]). Processed miRNAs are loaded into their main protein cofactor, Argonaute (AGO), which then associates with members of the GW182 family of proteins, forming the microRNA Induced Silencing Complex (miRISC). Mature miRISCs bind to the target messenger RNAs (mRNAs) and repress their translation and/or destabilize the target mRNA [3], [4].

RNA binding proteins (RBPs) make up another class of post-transcriptional gene regulators. RBPs can affect miRNA gene-repressive activity in a variety of ways, including through miRNA processing [5] and mRNA co-targeting through mRNA processing, transport, localization, stability/degradation and mRNA translation regulation. A given mRNA bound by miRISC also serves as a platform for binding of additional RNA-interacting factors, and a few have been shown to associate with core miRISC and modulate its activity [6], [7], [8]. For example, NHL-2 and CGH-1 physically interact with miRISC and enhance the repression of miRNA target genes [7], a process regulated by casein kinase II [9]. Furthermore, many genes, including RNA binding proteins (RBPs), have been identified as genetic interactors of the *let-7* family of miRNAs [10], [11], [12], [13]. It remains unclear how many of them function as direct modulators of miRISC activity. Complementary to these approaches, we have previously sought to identify physical interactors of ALG-1, a miRNA-specific *C*. *elegans* Argonaute [14], hypothesizing that proteins that co-precipitate with ALG-1 include factors that modulate miRNA-induced gene repression. An RNAi-based screen of the putative ALG-1 co-factors has identified HRPK-1, a conserved homolog of human heterogeneous nuclear ribonucleoprotein K (hnRNPK), as a novel miRNA interactor that modulates activity of several miRNAs.

Heterogeneous nuclear ribonucleoproteins (hnRNPs) form a large family of nucleic acid binding proteins with diverse functions in a wide range of cellular processes, including transcription, RNA processing, RNA transport, RNA stability, and translational repression [15]. They possess multiple domains and are thought to form modular complexes, increasing the diversity of the RNA/protein interactions [16]. Several hnRNPs have recently emerged as having roles in miRNA-mediated gene regulation [15]. Some are implicated in biogenesis of specific miRNAs [17], while others, such as hnRNP Q, appear to hinder miRNA activity by competing with key molecular effectors of miRISC [18]. KH domain proteins are a subclass of hnRNPs with similarly diverse functions [15]. These proteins contain the KH nucleic acid binding motifs, which were originally identified in hnRNPK, with KH domain named for K homology [19]. KH domains are evolutionary conserved and are found in approximately 40 human genes and 27 *C*. *elegans* genes. These nucleic acid binding motifs are 70 amino acids in size and can be present as a single domain or in multiple copies within a given protein [20], [19]. For example, Vigilin, a ribosome associated protein, contains 14 KH domains [21], [22]. It is thought that each KH domain can act as an independent binding module [23], although it is not currently clear if that holds true for all KH-containing proteins. Interestingly, a *C*. *elegans* homolog of the Vigilin gene, *vgln-1*, has been recently shown to cooperate with miRNAs to regulate gene expression [24].

Recent studies have implicated the human hnRNPK in miRNA-mediated gene repression. One study reports that hnRNPK may compete with multiple miRNAs for 3’UTR binding of their target gene, *PLK1* [25]. Such target binding competition puts hnRNPK in a role of a negative regulator of miRNA activity [25]. Other studies have placed hnRNPK in close physical proximity to miRNAs themselves, observing hnRNPK binding to miR-122 miRNA directly and potentially regulating its stability [26] or binding near miR-122 target sites on target mRNAs [27]. The functional significance of these interactions has not yet been described. In *C*. *elegans*, two KH domain-containing proteins, GLD-1 and VGLN-1 have been shown to genetically and/or physically interact with miRNA machinery [28] and [24], respectively. The mechanism through which GLD-1 and VGLN-1 functionally interact with the miRNA pathways remains unclear.

We have previously identified HRPK-1(F26B1.2), an hnRNPK homolog, in ALG-1 Argonaute immunoprecipitates as a putative ALG-1 physical interactor [14]. Here, we report the characterization of *hrpk-1* as a functional miRNA interactor that modulates activity of several miRNAs. Loss of *hrpk-1* results in a number of developmental defects, including sterility, embryonic lethality, vulval bursting, loss of alae, and abnormal gonad formation. Genetic loss of HRPK-1 enhances miRNA reduction-of-function phenotypes, consistent with HRPK-1 functional requirement for wild type miRNA activity. We report that HRPK-1 is ubiquitously expressed throughout *C*. *elegans* development and localizes to both nuclei and cytoplasm in some tissues, while remaining strongly nuclear in others. We confirm that HRPK-1 and ALG-1 co-precipitate. Finally, we find that *hrpk-1* may play a role in biogenesis of select miRNAs by promoting processing of some and inhibiting processing of others. HRPK-1 is not needed for ALG-1/AIN-1 miRISC assembly, suggesting that HRPK-1 may also functions at the level of miRNA target repression, especially in case of miRNAs whose processing does not depend on HRPK-1 activity.

Our data suggest that HRPK-1 may regulate miRNA activity by interacting with miRNA-associated protein complexes and modulating the efficacy of both mature miRNA processing and miRNA activity on target mRNAs. This furthers our understanding of how miRNAs may be regulated by or function in concert with RNA binding proteins and therefore integrate distinct developmental and physiological signals with miRNA-mediated gene repression.

## Results

**Fig 1:**
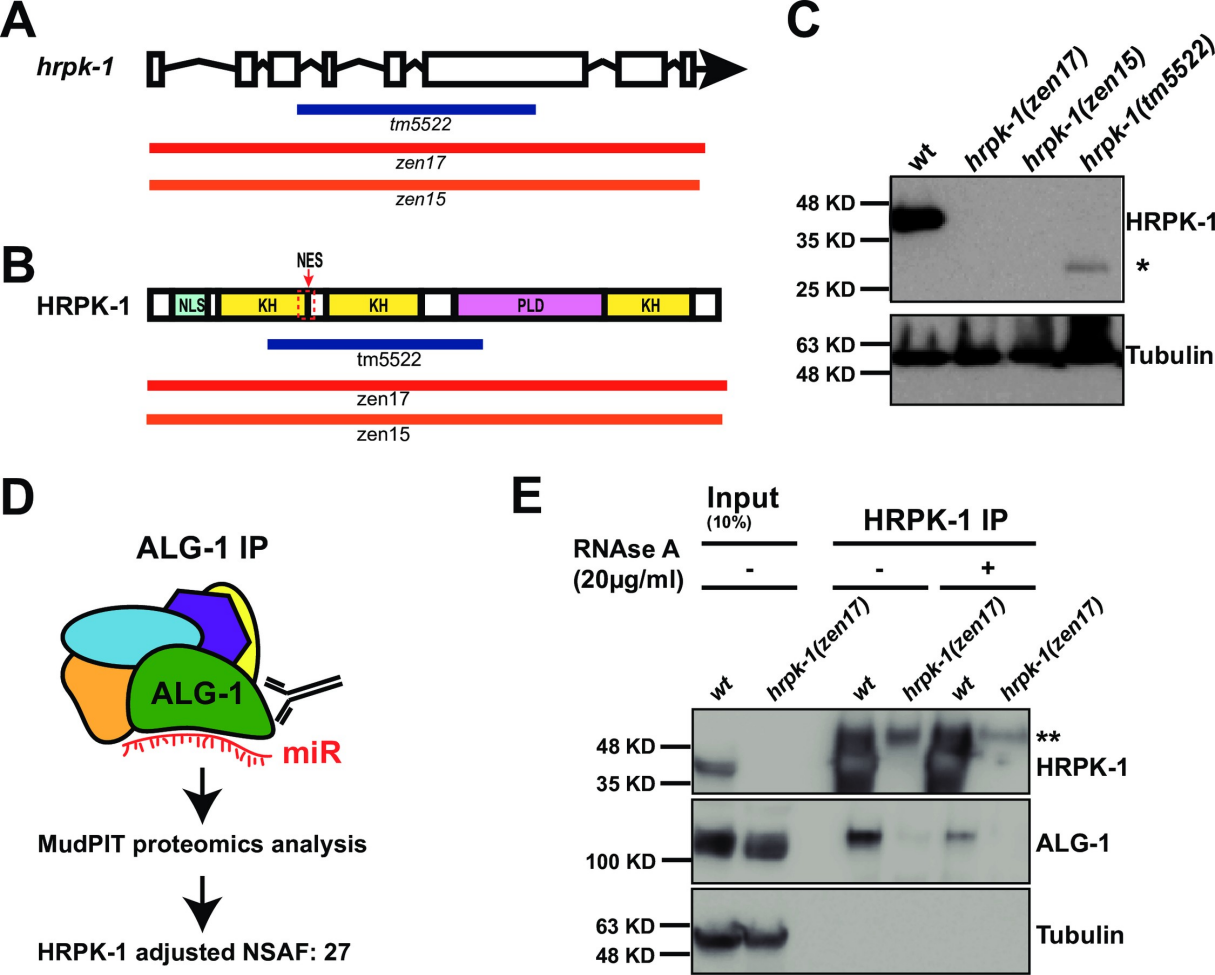
Multiple *hrpk-1* alleles produce deletions within the *hrpk-1* locus; ALG-1 co-precipitates with HRPK-1. (**A**) A schematic showing the predicted exon/intron structure of *hrpk-1* gene. *hrpk-1* alleles *tm5522*, *zen17*, and *zen15* delete parts or all of *hrpk-1*. (**B**) The *hrpk-1(tm5522)* allele is predicted to produce a truncated form of HRPK-1 protein, removing half of the first KH domain, second KH domain, and part of the PLD domain. NLS—predicted nuclear localization signal, KH—KH domain, NES—predicted nuclear export signal, PLD—prion like domain. (**C**) Western blotting for HRPK-1 protein detects the HRPK-1(tm5522) truncated protein at ~27kDA (highlighted by *). No protein is detected from the *hrpk-1(zen17)* and *hrpk-1(zen15)* null animals. Tubulin is detected as a loading control. (**D**) HRPK-1 was identified by MudPIT mass spectrometry in ALG-1 co-precipitates [14]. NSAF = normalized spectral abundance factor. (**E**) Western blotting for HRPK-1 and ALG-1 proteins in the HRPK-1 immunoprecipitates treated and untreated with RNAse A. Tubulin is detected as a loading control for input. Input = 10% of IP. **this non-specific band is most likely antibody heavy chain.

To identify and characterize potential physical and functional interactions of HRPK-1 with miRISC components, we first generated a null allele in *hrpk-1*. A previously existing deletion allele of *hrpk-1*, *hprk-1(tm5522)*, causes an in-frame deletion within the *hrpk-1* gene, resulting in production of a truncated HRPK-1 protein (Fig 1A–1C). Using CRISPR/Cas9-based genome editing, we generated two independent null alleles of *hrpk-1*, *hrpk-1(zen15)* and *hrpk-1(zen17)* (Fig 1A–1C). Both alleles nearly completely or completely delete the *hrpk-1* locus (Fig 1A) and produce no HRPK-1 protein (Fig 1B and 1C). Since both null alleles produce a similar loss-of function phenotype (S1 Fig), we have designated the larger deletion, *hrpk-1(zen17)*, as the reference null allele for *hrpk-1* and used it in subsequent analyses.

### ALG-1 argonaute and HRPK-1 co-immunoprecipitate in a partially RNA dependent manner

We have previously identified HRPK-1(F26B1.2) by immunoprecipitating *C*. *elegans* miRNA-specific Argonaute ALG-1 and analyzing the co-purified protein complexes by MudPIT proteomics [14], (Fig 1D). To confirm this physical interaction, we performed the reciprocal IP using antisera generated against HRPK-1 and probed for the presence of ALG-1. We found that HRPK-1 IP co-precipitates ~2–3% of ALG-1 (Fig 1E), consistent with HRPK-1 interaction with miRNA-associated machinery.

To address the possibility of a spurious interaction due to the abundant nature of hnRNP proteins, we tested the ability of ALG-1 to co-precipitate another abundant hnRNP protein and hnRNPA1 homolog, HRP-1. We found that HRP-1 fails to precipitate with ALG-1 despite its abundance (S2 Fig), suggesting that ALG-1/HRPK-1 interaction is specific.

To determine whether HRPK-1/ALG-1 co-immunoprecipitation is RNA dependent, we performed the HRPK-1 IP in the presence of RNAse A (20μg/ml). Interestingly, incubation of lysates with RNAse A prior to and during HRPK-1 IP reduces but does not abolish ALG-1 co-precipitation with HRPK-1 (Fig 1E). This result suggests that while the ALG-1/HRPK-1 binding may in part depend on RNA, the two proteins may also be interacting directly, rendering the observed interaction partially RNAse A resistant (Fig 1E). ALG-1/HRPK-1 interaction may be strengthened through RNA-protein association, but at this point we cannot rule out the possibility that HRPK-1/ALG-1 interaction is RNA dependent.

### *hrpk-1* is required for a number of developmental processes

**Fig 2:**
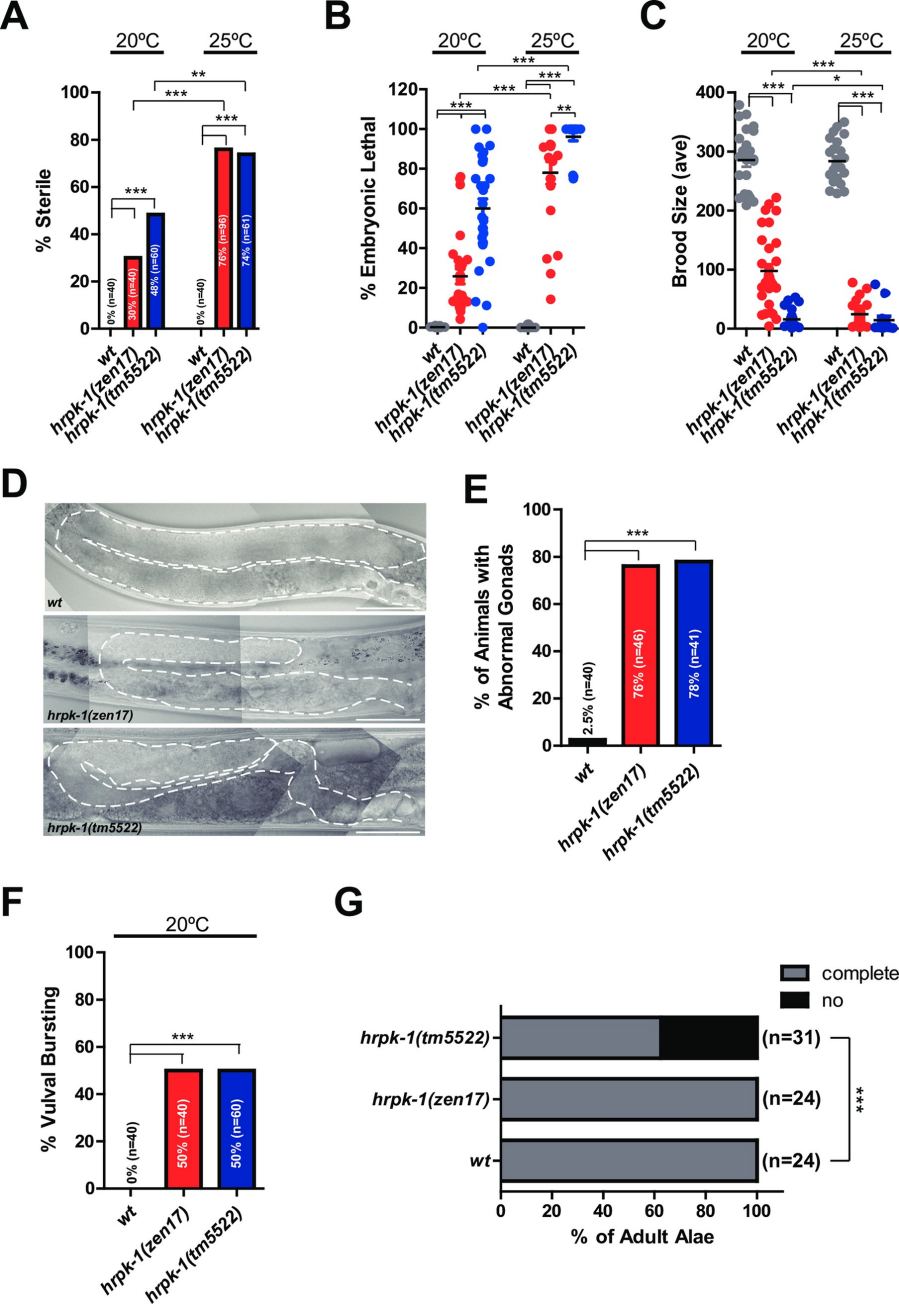
*hrpk-1* mutations cause developmental defects at 20°C and 25°C. *hrpk-1(zen17)* and *hrpk-1(tm5522)* mutations result in temperature sensitive animal sterility (**A**), embryonic lethality (**B**), and reduced brood size (**C**). *hrpk-1(zen17)* and *hrpk-1(tm5522)* mutations cause abnormal gonad formation (**D**, quantified in **E**), vulval bursting in day 3 or older adults (**F**), and defects in young adult alae formation (**G**). Bar in (**D**) = 50μm. Chi-square was used to analyze phenotypic data shown in A, E-G, T-test was used to analyze embryonic lethality (B), and F-test was used to analyze brood size (C). *p≤0.*05*, **p≤0.*01*, ***p≤0.*001*.

To determine the effects of *hrpk-1* mutations on animal development, we characterized gross morphological phenotypes of both *hrpk-1(tm5522)* and *hrpk-1(zen17)* mutants. We found that both alleles induce temperature sensitive sterility (Fig 2A), embryonic lethality (Fig 2B), and reduced brood size (Fig 2C). Both alleles also cause gonad formation defects (Fig 2D and 2E) and vulval bursting in day 3 or older adults (Fig 2F). We observed that almost in every instance, homozygous *hrpk-1(tm5522)* mutants exhibit more severe phenotypes than complete loss of *hrpk-1* (Fig 2A–2C and 2G), with the exception of gonad morphology (Fig 2D and 2E) and vulval bursting (Fig 2F) defects. *hrpk-1(tm5522)* mutant animals also fail to produce adult alae approximately 40% of the time (Fig 2G). In addition, *hrpk-1* function appears to be maternally required for fertility (S3A Fig), brood size (S3B Fig), and embryonic viability (S3C Fig). Specifically, *hrpk-1(zen17) [m-z-]* animals have increased defects compared to *hrpk-1(zen17) [m+z-]* in sterility (S3A Fig), brood size (S3B Fig), and embryonic lethality (S3C Fig).

### *hrpk-1(tm5522)* allele is antimorphic

Our genetic analysis suggests that *hrpk-1(tm5522)* allele is weakly semi-dominant and antimorphic in nature (S3 Fig). Firstly, homozygous *hrpk-1(tm5522)* mutants almost always exhibit more severe phenotypes than *hrpk-1(zen17)* null animals (Fig 2A–2C and 2G). Secondly, *hrpk-1(tm5522)/+ [m-z+]* animals carrying only a single copy of the wild type allele exhibited higher fertility defects and vulval bursting than *hrpk-1(zen17)/+ [m-z+]* animals (14% sterility versus 0%, respectively, S3A and S3D Fig). Thirdly, *hrpk-1(tm5522)/+ [m+z+]* animals show a modest reduction in brood size (S3B Fig) and embryonic lethality (S3C Fig) and vulval bursting (S3D Fig) not normally observed in wild type animals. Furthermore, animals carrying a single copy of maternally provided *hrpk-1(tm5522)* allele (*hrpk-1(tm5522)*⚥*/hrpk-1(zen17)*♂ produced by *hrpk-1(tm5522)* mothers and *hrpk-1(zen17)* fathers) exhibit higher rates of sterility (S3A Fig), have smaller brood sizes (S3B Fig), and higher embryonic lethality (S3C Fig) than *hrpk-1(zen17) [m-z-]* animals lacking functional *hrpk-1* completely. Animals carrying a single copy of maternally provided *hrpk-1(tm5522)* allele (*hrpk-1(tm5522)*⚥*/hrpk-1(zen17)*♂also show more severe phenotypes than *hrpk-1(tm5522)*♂/ *hrpk-1(zen17)*⚥ animals produced by *hrpk-1(tm5522)* fathers and *hrpk-1(zen17)* mothers (S3A–S3C Fig), likely a result of both the antimorphic nature of the *tm5522* allele and the maternal component to its function. Overall, while the genetic analysis is somewhat complex due to the maternal requirement for *hrpk-1* activity, taken together, these genetic data support the antimorphic, weakly semi-dominant nature of the *hrpk-1(tm5522)* allele.

### *hrpk-1* functionally interacts with the *let-7 family* of miRNAs

**Fig 3:**
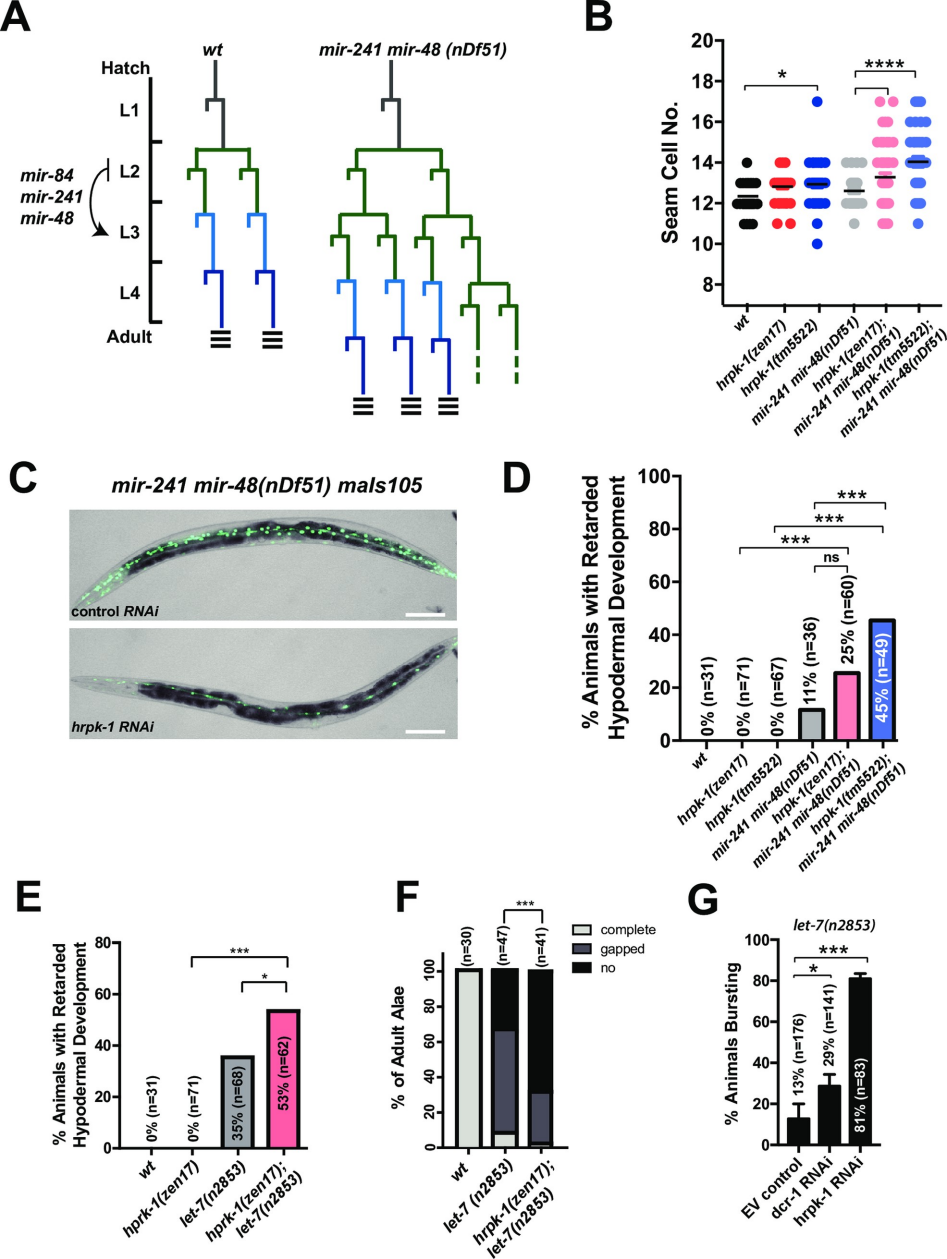
*hprk-1* functionally interacts with the *let-7* family of miRNAs. (**A**) Schematic seam cell lineages of wild type and *mir-241 mir-48* mutant animals throughout larval development. (**B**) *hrpk-1* mutations increase the seam cell numbers in *mir-48 mir-241(nDf51)* young adults. (**C**) Loss of wild type *hrpk-1* activity enhances the retarded hypodermal *col-19*::*gfp* expression phenotype of *mir-48 mir-241(nDf51)* mutants (quantified in (**D**)). *hrpk-1(zen17)* enhances the retarded (**E**) alae formation phenotype and (**F**) *col-19*::*gfp* expression observed in *let-7(n2853)* animals at 20°C. (**G**) RNAi knockdown of *hrpk-1* enhances vulval bursting of *let-7(n2853)* animals at 15°C. Animals scored in (**B**-**E**) carry the *col-19*::*gfp(maIS105)* transgene. All animals in (**E, F**) carry *lin-2(e1309)*, which suppresses the vulval bursting of *let-7(n2853)* animals through a non-heterochronic mechanism. Bar in (**C**) = 100μm. T-test was used to analyze seam cell number (B) and vulval bursting (G) and chi-square was used to analyze all other phenotypic data. *p≤0.*05*, **p≤0.*01*, *** p≤0.*001*, **** p≤0.*0001*.

To test whether *hrpk-1* functions in miRNA-dependent cell fate specification, we examined potential genetic interactions between *hrpk-1* and *let-7* family of miRNAs that control temporal cell fate specification throughout larval development. The *let-7* miRNAs regulate the stage specific cell gene expression programs in a number of tissues, with the *let-7* family mutant phenotypes most evident in the *C*. *elegans* seam and hypodermis (for review, see [29]). Specifically, *let-7* family miRNAs, together with other heterochronic genes, regulate cell division patterns of the seam cells during larval development [30] and seam cell terminal differentiation at the transition from larval development to adulthood [31]. *mir-48*, *mir-241*, and *mir-84*, three members of the *let-7* miRNA family, are transcriptionally up-regulated in the late L1 stage and subsequently down-regulate the expression of the *hbl-1* transcription factor during the L2 stage [30]. As a consequence of these regulatory interactions, *mir-48*, *mir-241*, and *mir-84* limit the proliferative seam cell division pattern of hypodermal stem cells to the L2 stage and promote subsequent L3-associated patterns of seam cell divisions [30], (Fig 3A). *mir-48*, *mir-241*, and *mir-84* are genetically redundant, with single and double mutants of these microRNAs exhibiting partially penetrant cell retarded heterochronic phenotypes [30]. These phenotypes can be monitored by observing alterations in seam cell lineage as well as defects associated with a reduction in the expression of adult-specific reporters (*col-19*::*gfp*) after the 4^th^ larval stage [30,32], (Fig 3B and 3C). *hrpk-1(zen17)* enhances the retarded phenotype of *mir-48 mir-241 (nDf51)* mutants (Fig 3B–3D). Specifically, loss of *hrpk-1* activity enhances the retarded expression of adult hypodermal marker, *col-19*::*gfp(maIs105)*, (Fig 3C and 3D) and results in an increased number of seam cells as compared to *mir-48 mir-241(nDf51)* alone (Fig 3B). This enhancement of the *mir-48 mir-241(nDf51)* heterochronic phenotype by loss of *hrpk-1* is consistent with *hrpk-1* functional requirement for efficient activity of the remaining intact *let-7* family miRNAs.

*let-7*, the founding member of the *let-7* family of miRNAs, controls terminal cell fate specification, which occurs during the developmental progression through the late larval stages and into the adulthood [31]. A temperature sensitive mutation that compromises but does not completely abolish let-7 activity, *let-7(n2853)*, causes a retarded development phenotype [31], [33]. *let-7(n2853)* animals have abnormal/absent alae in young adults, display delayed hypodermal expression of the adult marker *col-19*::*gfp* at 20°C (Fig 3E and 3F), and rupture through the vulva during the L4-adult molt [31]. *hrpk-1* knockout enhances retarded *col-19*::*gfp(maIS105)* expression (Fig 3E) and the retarded alae phenotype (Fig 3F) observed in the *let-7(n2853)* mutants. Loss of *hrpk-1* alone is not enough to induce a heterochronic phenotype (Fig 3A, 3D and 3C). Knockdown of *hrpk-1* by RNAi enhances the vulval rupture phenotype of *let-7(n2853)* animals at 15°C (Fig 3G). The observed enhancement of the *let-7(n2853)* retarded phenotype by *hrpk-1* mutations is consistent with the hypothesis that *hrpk-1* function is important for *let-7* miRNA activity.

### *hrpk-1* functionally interacts with the *lsy-6* miRNA and is required for efficient regulation of *lsy-6* target *cog-1*

**Fig 4:**
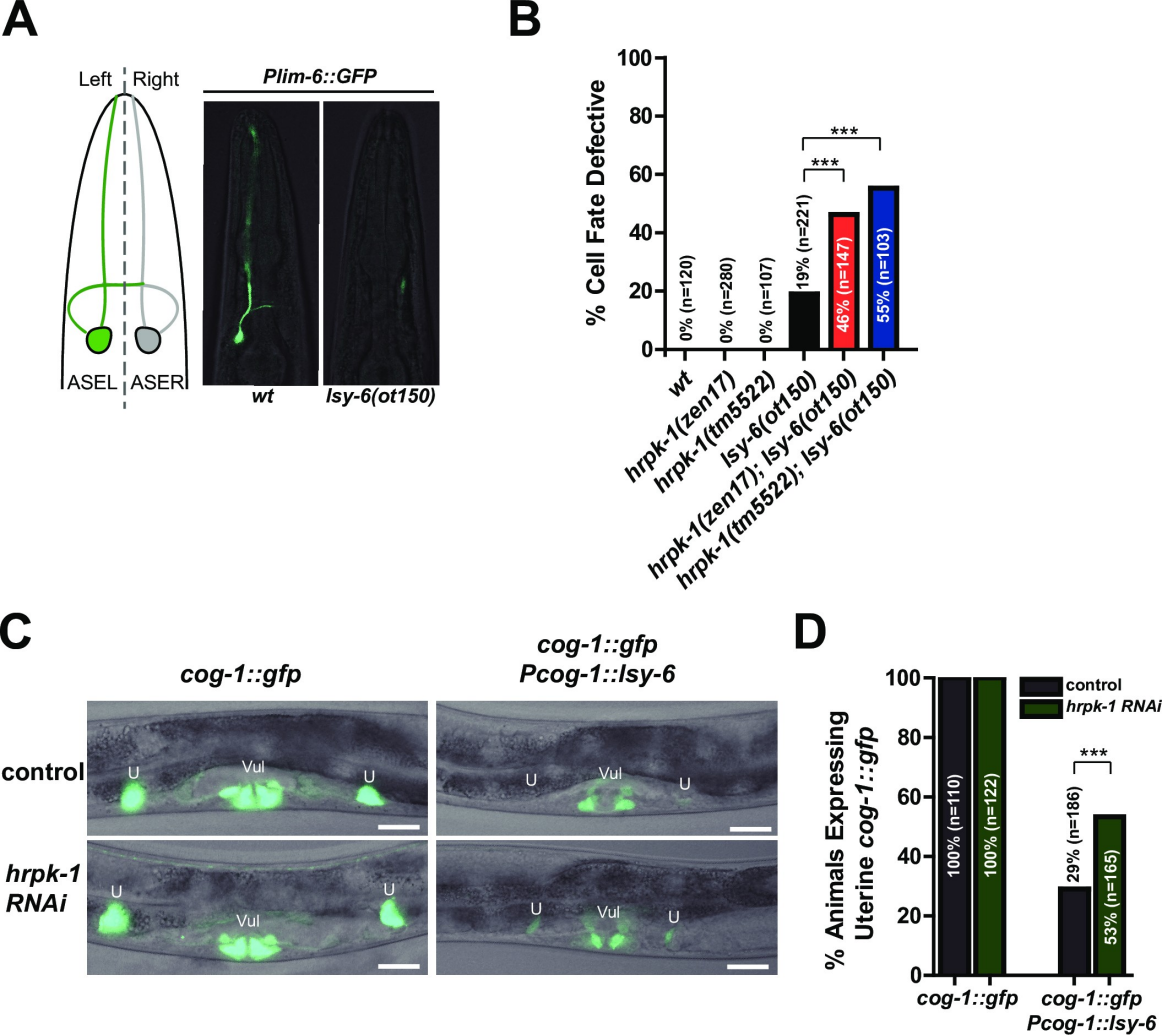
*hrpk-1* function is required for efficient *lsy-6* miRNA activity. *Plim-6*::*gfp* reporter marks the ASEL cell fate (**A**). The low penetrance defective cell fate phenotype observed in *lsy-6(ot150)* animals is enhanced by *hrpk-1* mutations (**B**). (**C**) *hrpk-1* RNAi relieves the lsy-6-mediated repression of *cog-1*::*gfp* in the uterine cells, quantified in (**D**). Animals in (B) also carry *Plim-6*::*gfp* reporter. Bar in (C) = 20μm. Chi-square, ***p<0.*001*.

To test the hypothesis that HRPK-1 may be required for miRNA activity regulation, we looked for the effects of *hrpk-1* mutations on the activity of *lsy-6* miRNA-dependent processes. *lsy-6* regulates cell fates of two bilaterally symmetrical ASE neurons [34]. ASEL-specific expression of *lsy-6* down-regulates its key target, *cog-1*, while uninhibited *cog-1* expression within the ASER dictates that neuron’s cell fate [34]. The ASEL cell fate is distinguished by the expression of a downstream reporter, *Plim-6*::*gfp*, an established marker for the ASEL cell fate [34], [35], (Fig 4A). Reduction of lsy-6 activity through a cis-regulatory mutation in the *lsy-6* promoter, *lsy-6(ot150)*, causes a low penetrance phenotype where the ASEL neuron adopts the cell fate of ASER approximately 20% of the time [36], (Fig 4A and 4B). To assess *hrpk-1-lsy-6* functional interaction, we removed *hrpk-1* in the presence of the reduction of function *lsy-6(ot150)* allele. Genetic mutations in *hrpk-1* significantly enhance the cell fate defective phenotype observed in the *lsy-6(ot150)* animals (Fig 4B). *hrpk-1* mutations alone are not sufficient to induce an ASEL to ASER cell fate switch (Fig 4B). Importantly, *hrpk-1* RNAi relieves the lsy-6-mediated inhibition of its target, *cog-1*::*gfp*, in uterine cells (Fig 4C and 4D), suggesting that HPRK-1 is required for efficient inhibition of *cog-1* by lsy-6 miRNA in that tissue.

### *hrpk-1* functionally interacts with the *mir-35-42* family of miRNAs

**Fig 5:**
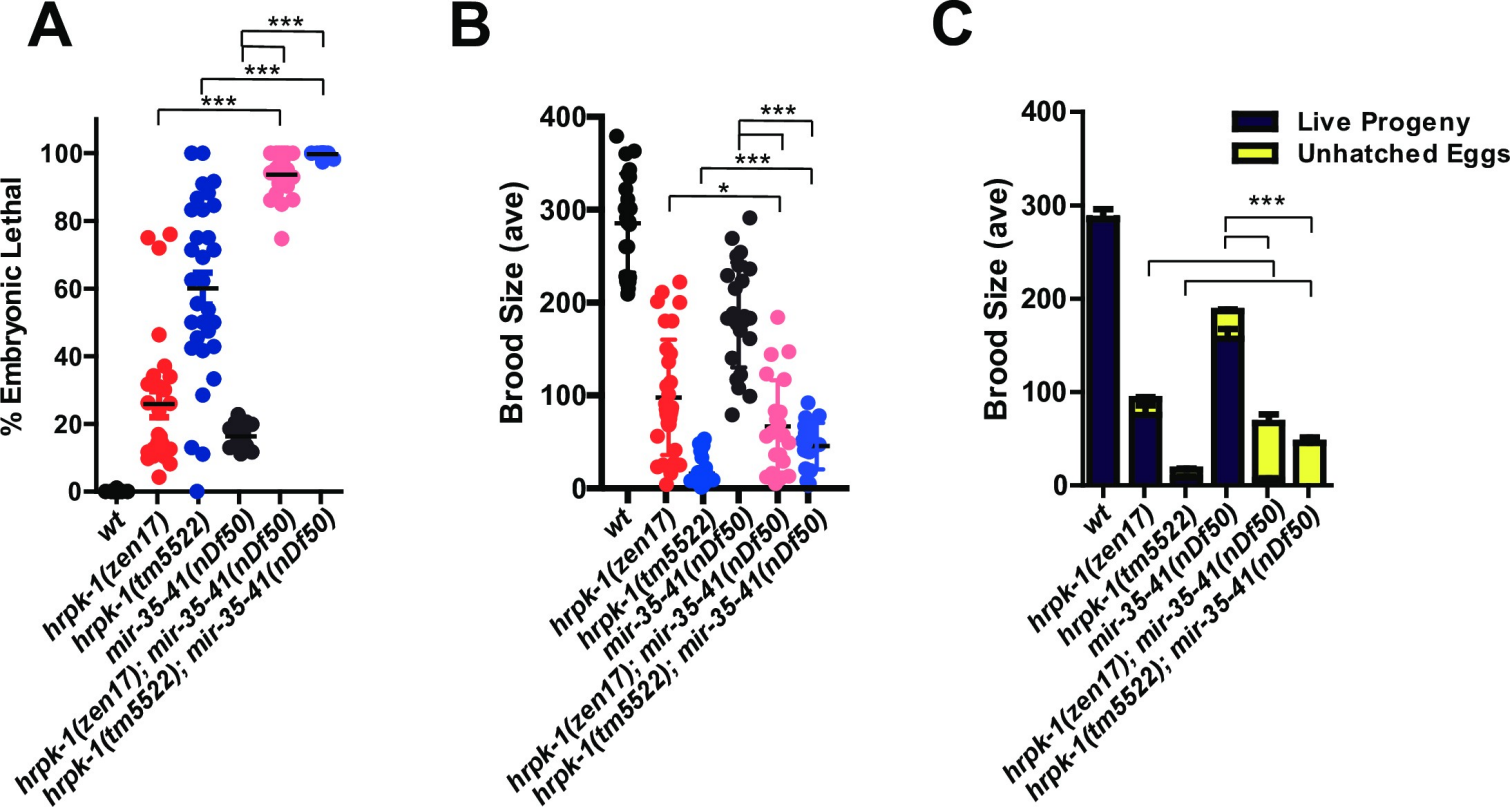
*hrpk-1* mutations enhance *mir-35-41(nDf51)* mutant phenotype at 20°C. *hprk-1* mutations enhance *mir-35-41(nDf51)* embryonic lethality (**A**) and further reduces *mir-35-41(nDf51)* brood sizes (**B-C**). (**C**) shows the brood breakdown by live and dead progeny. T-test (A), F-test (B), Chi-square (C) were used for statistical analysis, *p<0.*05* and ***p<0.*001*.

To assess whether *hrpk-1* may be broadly required for miRNA activity, we reduced its function in other miRNA sensitized backgrounds. *miR-35-42* family of miRNAs controls fertility and embryonic development of *C*. *elegans* [37]. Loss of all members of the *miR-35* miRNA family results in fully penetrant sterility and embryonic lethality phenotypes [37]. Deletion of a genomic region harboring 7 of the 8 miRNAs, *mir-35-41(nDf50)*, causes animals to exhibit a temperature sensitive increase in embryonic lethality and overall reduction of brood size [37], [38], (Fig 5A and 5B). Combining either of the *hrpk-1* deletions *(hrpk-1(zen17)* or *hrpk-1(tm5522))* with *mir-35-41(nDf50)* results in a strong synthetic lethal phenotype where a majority of animals fail to develop (Fig 5A). This genetic synergy in combined mutants is also recapitulated in a dramatic decrease in overall brood size (Fig 5B) in animals reared at the semi-permissive temperature of 20°C. *hrpk-1(tm5522)* causes a greater enhancement of the *mir-35-41(nDf50)* phenotype than *hrpk-1(zen17)* null (Fig 5A and 5B), consistent with the antimorphic nature of the *hrpk-1(tm5522)* allele. Importantly, the enhancement of the *mir-35-41(nDf50)* phenotype by *hrpk-1* mutations is not simply additive, but synergistic (Fig 5), consistent with the hypothesis that *hrpk-1* is required for activity of the remaining miRNA, miR-42.

### HRPK-1 is ubiquitously expressed throughout *C*. *elegans* development.

**Fig 6:**
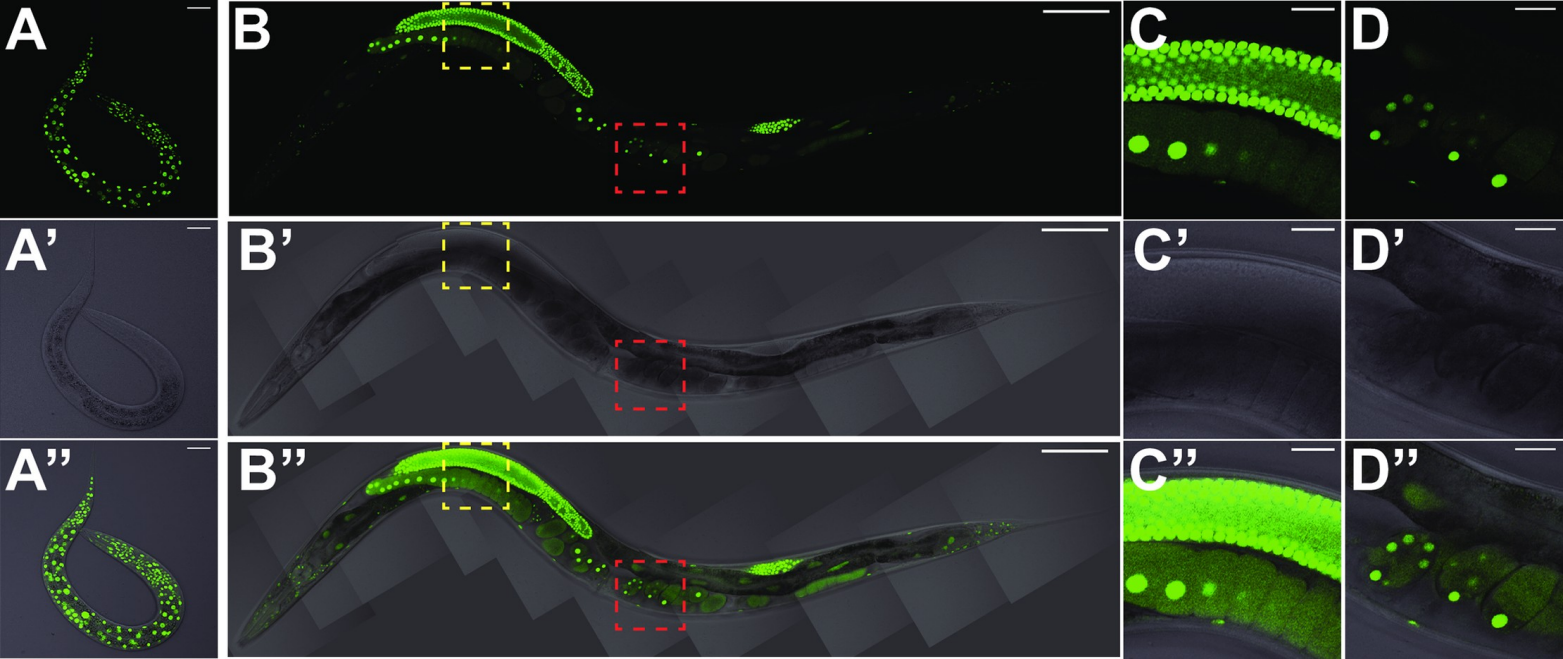
Endogenously tagged *hrpk-1*::*gfp* transgene *(hrpk-1(zen64))* is expressed throughout *C*. *elegans* development. (**A**) L1 larva shows ubiquitous HRPK-1::GFP expression in the nuclei of somatic tissues and the germline precursor cells. (**B**) HRPK-1::GFP fluorescence in an adult animal highlights ubiquitous HRPK-1 expression in somatic tissues and in the animal’s germline. Zoom in micrographs of (**B**) highlight strong nuclear and cytoplasmic expression in the germline (**C**) and early embryos (**D**). (**A’-D’**) DIC images of (**A-D**). (**A”-D”**) merged images of fluorescent micrographs (**A-D**) and the DIC images (**A’-D’**). Bar in (**A**), (**C**), (**D**) = 20μm, bar in (**B**) = 100μm.

To gain insight into HRPK-1 function, we characterized both spatial and temporal expression of endogenous HRPK-1 using a CRISPR-generated *hrpk-1*::*gfp* transgene (Fig 6). *hrpk-1*::*gfp* is ubiquitously expressed during all *C*. *elegans* developmental stages (Fig 6A–6D). The C-terminally tagged *hrpk-1*::*gfp* expression is observed in the gut, muscle, neuronal, and hypodermal tissues, where it localizes to the cell nuclei (Fig 6A and 6B). Interestingly, *hrpk-1*::*gfp* is strongly present in the animal germline, oocytes, and early embryos, where its subcellular localization is both nuclear and cytoplasmic (Fig 6B–6D). The subcellular localization of HRPK-1::GFP is consistent with the predicted nuclear localization and nuclear export signals found within the *hrpk-1* sequence (Fig 1B). Our inability to detect *hrpk-1*::*gfp* signal in the cytoplasm of somatic cells may reflect HRPK-1 distinct functions between the soma and the germline or may simply be due to a lower cytoplasmic expression level that falls below our detection limits. In fact, HRPK-1 somatic cytoplasmic localization has been observed in large scale subcellular proteome mapping [39], suggesting that we may be limited in detecting HRPK-1 in the cytoplasm in the presence of strong nuclear expression. Overall, the ubiquitous spatial and temporal *hrpk-1* expression is consistent with the apparent *hrpk-1* roles in a number of developmental processes, including embryonic and larval development, as well as fertility.

### Loss of *hrpk-1* affects levels of select miRNAs but does not affect ALG-1/AIN-1 miRISC assembly

**Fig 7:**
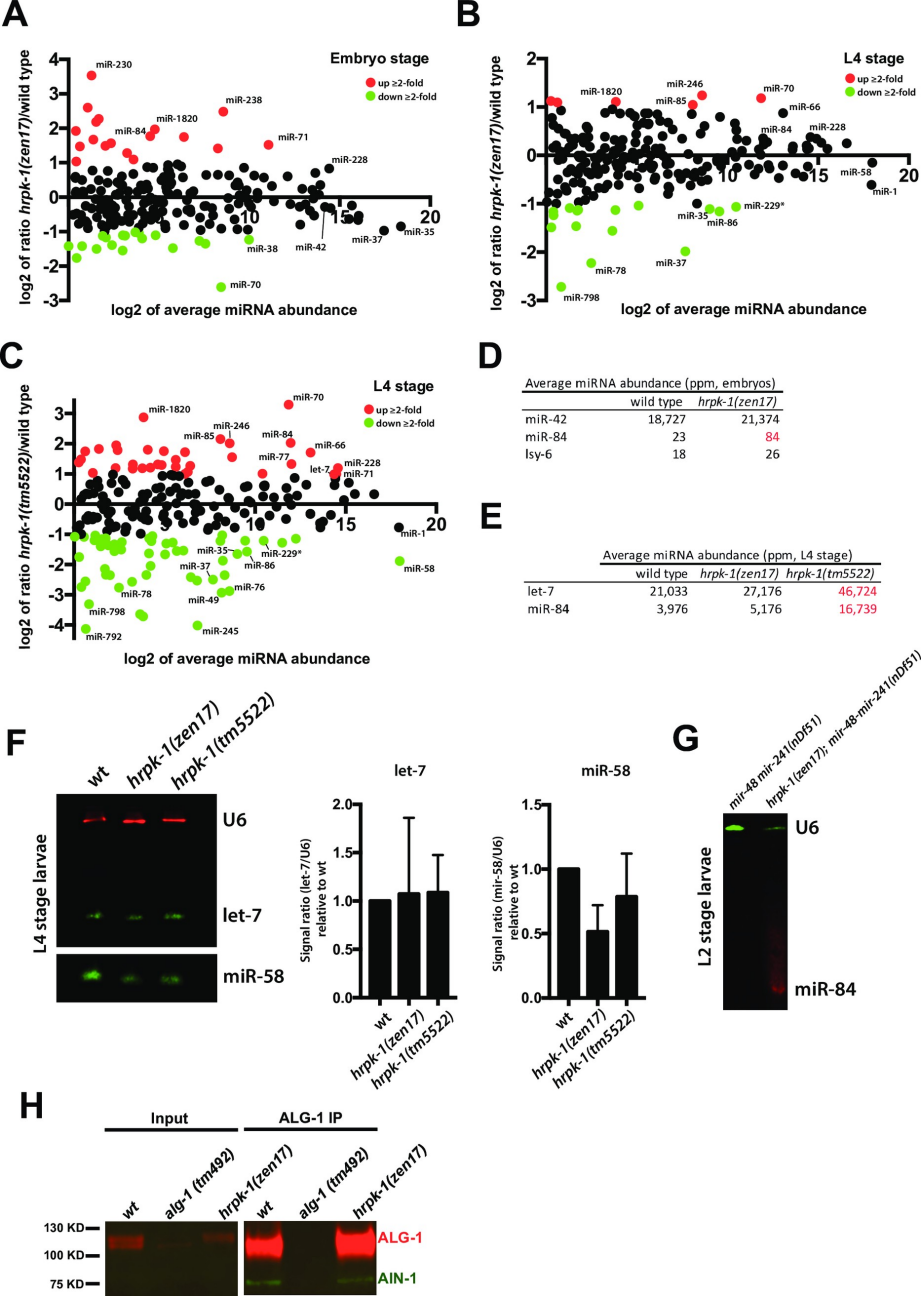
miRNA abundances and miRISC formation in *hrpk-1* mutant animals. Mature miRNA abundances were assayed in wild type and *hrpk-1* mutants by small RNAseq analysis (**A-E**). Scatterplots showing log fold change in miRNA abundance between wild type and *hrpk-1(zen17)* embryos (**A**), wild type and *hrpk-1(zen17)* L4 larvae (**B**), and wild type and *hrpk-1(tm5522)* L4 larvae (**C**). Average miR-42, miR-84, and lsy-6 reads in wild type and *hrpk-1(zen17)* embryos (**D**) and average let-7 and miR-84 reads in wild type and *hrpk-1* mutant L4 larvae (**E**), with statistically significant changes in read numbers highlighted in red. (**F**) let-7 and miR-58 Northern blot and signal quantifications across three biological replicates as normalized to wild type miRNA/U6 signal ratio; L4 stage larvae RNA. (**G**) Representative miR-84 Northern blot using L2 stage RNA from *mir-48 mir-241(nDf51)* and *hrpk-1(zen17); mir-48 mir-241(nDf51)* animals. (**H**) Western blot of ALG-1 IP shows that AIN-1 co-precipitates with ALG-1 in *hrpk-1* mutant animals to levels similar to wild type.

**Fig 8:**
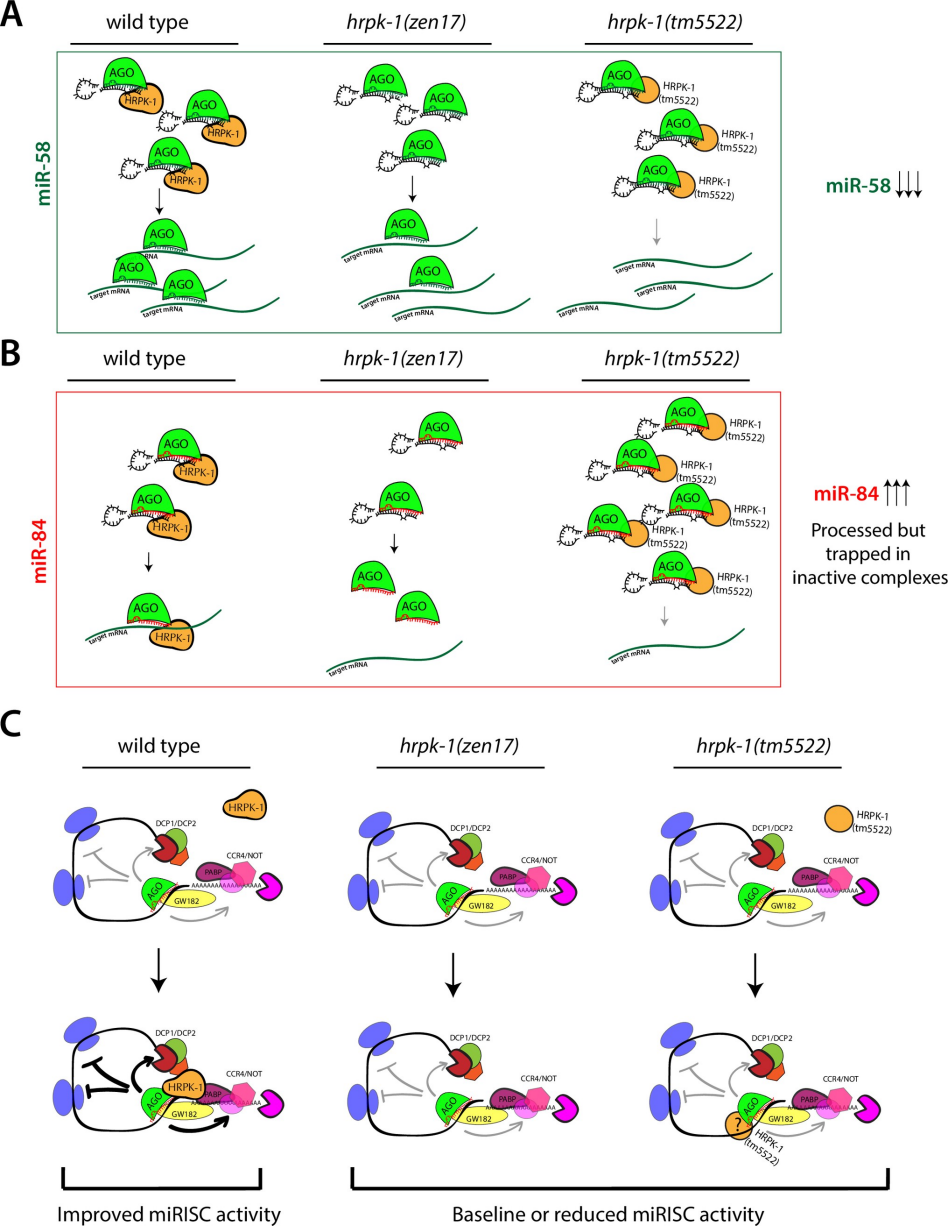
Models representing potential mechanisms through which HRPK-1 may affect miRNA activity. For miRNAs whose abundance is affected by *hrpk-1* mutations, HRPK-1 may cooperate with miRLC and participate in miRNA biogenesis by either enhancing (**A**) or restricting (**B**) specific miRNA processing. For other miRNAs, HRPK-1 may bind mRNAs near miRNA target sites and enhance miRISC::mRNA target interaction through miRISC binding (**C**). In this model, HRPK-1 may also increase miRISC activity through augmenting miRISC interaction with downstream effector complexes.

Functional regulation of the miRNA activity by HRPK-1 could occur at the level of miRNA processing, miRISC assembly, or miRISC activity. To determine whether HRPK-1 is necessary for miRNA biogenesis, we cloned and sequenced miRNAs from wild type, *hrpk-1(zen17)*, and *hrpk-1(tm5522)* L4 larvae and wild type and *hrpk-1(zen17)* embryos (S1 Table). We found that loss of *hrpk-1* affects levels of some mature miRNAs, with specific miRNA species increasing, and some decreasing, in abundance (Fig 7A–7E, S1–S4 Tables). This pattern was observed in *hrpk-1(zen17)* embryos (Fig 7A, S2 Table) and L4 larvae (Fig 7B, S3 Table). Interestingly, the changes in miRNA abundances were more pronounced in the *hrpk-1(tm5522)* mutant animals, with more miRNAs affected by the antimorphic allele (Fig 7C, S4 Table). Additionally, levels of some miRNAs were altered more significantly in *hrpk-1(tm5522)* mutants (Fig 7C) compare to *hrpk-1(zen17)*, (Fig 7B). For example, levels of mature miR-70 were 2-fold higher in *hrpk-1(zen17)* L4 larvae (Fig 7B) and 8-fold higher in *hrpk-1(tm5522)* (Fig 7C) compared to their wild type counterparts (S3 and S4 Tables). Mature miR-86 levels were 2-fold lower in *hrpk-1(zen17)* (Fig 7B) and 3-fold lower in *hrpk-1(tm5522)* (Fig 7C) compared to wild type (S3 and S4 Tables). Interestingly, let-7 and miR-84 levels were slightly but not significantly increased in *hrpk-1(zen17)* animals (Fig 7B and 7E), but were significantly more abundant in *hrpk-1(tm5522)* mutants (Fig 7C and 7E) at the L4 stage (S1 Table). To visualize these changes, we performed infrared (IR) Northern blots for let-7 and an abundant miRNA, miR-58, which showed a slight decrease in levels in *hrpk-1(zen17)* animals (Fig 7B) and a significant decrease in *hrpk-1(tm5522)* mutants (Fig 7C). Even with the less quantitative nature of Northern blotting, we found that the trends displayed by these miRNAs were similar to those observed by small RNA sequencing (Fig 7F).

To assay miRNA levels in the mutant backgrounds used for our functional studies at stages relevant for their activity, we probed for miR-84 in *mir-48 mir-241(nDf51)* and *hrpk-1(zen17); mir-48 mir-241(nDf51)* L2 larvae. We observed that while miR-84 levels were below detection by our Northern method in *mir-48 mir-241(nDf51)*, more miR-84 was observed in *hrpk-1(zen17); mir-48 mir-241(nDf51)* animals (Fig 7G), suggesting that the absence of *hrpk-1* is responsible for mature miR-84 increase.

Based on these data we conclude that HRPK-1 contributes to biogenesis of a subset of the miRNAs by promoting processing of some and inhibiting processing of others. However, levels of many miRNAs, including miR-42 and lsy-6, were not significantly affected by *hrpk-1* mutations (Fig 7A–7D, S1 and S2 Tables), suggesting either that HRPK-1 function is not required for biogenesis of these miRNAs or that its role in miRNA processing is spatially or temporally restricted and is below our capability to detect these changes.

Mature miRISC formation involves Argonaute association with GW182 homologs [4]. To assess whether HRPK-1 may be needed for miRISC assembly, we tested the ability of ALG-1 to co-precipitate with its miRISC co-factor and GW182 homolog AIN-1 in *hrpk-1(zen17)*. We found that AIN-1 co-precipitates with ALG-1 in *hrpk-1* mutant animals at levels similar to those observed in wild type (Fig 7H). We did not test for ALG-2 and AIN-2 containing miRISC formation and therefore cannot rule out the possibility that HRPK-1 may be necessary for formation of miRISCs that include ALG-2 and/or AIN-2 proteins. However, we can conclude that HRPK-1 is not required for ALG-1/AIN-1 interaction as observed by our ALG-1 immunoprecipitation experiments (Fig 7H).

## Discussion

### HRPK-1 physically and functionally interacts with miRNA pathways

In this manuscript we report characterizations of *hrpk-1*, which encodes an RNA binding protein HRPK-1. HRPK-1 was originally identified by MudPIT proteomics in ALG-1 co-precipitates [14], (Fig 1D). Here, we confirm that HRPK-1 co-precipitates ALG-1 in a reciprocal experiment and show that the interaction between HRPK-1 and ALG-1 may be partially RNA-dependent (Fig 1E). The relatively small amount of ALG-1 (~2–3%) co-precipitating with HRPK-1 (Fig 1E) could reflect instability of complexes in our assay or be due to the many dynamic binding partners of both ALG-1 and HRPK-1. It is important to point out that in addition to HRPK-1 homology with human hnRNP K, the interaction between Argonaute and HRPK-1 also appears to be conserved as hnRNP K has previously been co-purified with three of four human AGO proteins [40].

In addition to confirming the conserved physical interaction between HRPK-1 and ALG-1, we report that *hrpk-1* is required for efficient activity of several miRNAs and miRNA families: *lsy-6*, *let-7-family*, and *mir-35-family*. In addition to enhancing the cell fate defects observed in *lsy-6(ot150)* animals, HRPK-1 promoted lsy-6 mediated repression of the lsy-6 target gene, *cog-1* (Fig 4C and 4D). Loss of *hrpk-1* did not cause a heterochronic phenotype normally observed in *let-7* family mutants (Fig 3), although the antimorphic *hprk-1(tm5522)* allele did produce a mild alae defect (Fig 2G) and an increased seam cell number (Fig 3B). Together, these data suggest that HRPK-1 is not an essential miRNA co-factor, but rather positively modulates *lsy-6* and *let-7* family miRNA activity. The synergistic genetic interaction between *hrpk-1* mutations and *mir-35-41(nDf50)* is also consistent with a hypothesized role for *hrpk-1* as a positive co-factor of miR-42 activity. Overall, we show that HRPK-1 physically interacts ALG-1, a major component of miRNA loading complex (miRLC) and miRISC, and functionally interacts with several miRNAs in a number of developmental processes.

### *hrpk-1* subcellular localization

The ubiquitous expression of *hrpk-1* is consistent with its pleotropic effects on *C*. *elegans* development. We observed a strong nuclear expression of endogenous *hrpk-1* (Fig 6) and both nuclear and cytoplasmic HRPK-1::GFP localization in the germline, oocytes, and early embryos. This is not surprising as HRPK-1 does contain a predicted nuclear localization signal and a predicted nuclear export signal (Fig 1B). Despite our inability to definitively detect HRPK-1::GFP in the cytoplasm of somatic tissues, a recent report mapping the subcellular-specific proteomes in a number of *C*. *elegans* tissues places HRPK-1 in the hypodermal cytoplasm using a proximity ligation dependent assay [39]. This assay may be able to capture transient protein shuttling and likely has a higher sensitivity than our fluorescent reporter observations. We reason that the observed steady-state nuclear HRPK-1::GFP localization does not mean there is no function for HRPK-1 in the cytoplasm. In fact, given the biochemical interaction between ALG-1 and HRPK-1, we hypothesize that cytoplasmic HRPK-1 activity may in fact be required for its functional interaction with the miRNA pathways. It remains to be seen whether nuclear HRPK-1 localization or movement between the nucleus and the cytoplasm plays a role in miRNA-dependent gene regulation.

### HRPK-1 role in miRNA gene regulatory activity

Several molecular models can explain the functional and physical interactions observed between HRPK-1 and miRNA machinery, with modulation of miRNA activity by HRPK-1 occurring at the level of miRLC or miRISC. Careful analysis of miRNA abundances at specific stages of *C*. *elegans* development revealed that loss of wild type HRPK-1 activity resulted in a decrease of mature miRNA levels for some miRNAs. Interestingly, the decrease in miRNA abundances were more significant in the *hrpk-1(tm5522)* mutants, correlating with the more severe phenotypes caused by this allele. For these miRNAs, such as miR-58, HRPK-1 may normally promote biogenesis (Fig 8A), with loss of HRPK-1 resulting in decreased processing and subsequent reduction in the levels of mature miRNAs. In this case, HRPK-1(tm5522) may prevent miRNA processing by trapping the necessary components in inactive complexes (Fig 8A), further reducing the levels of mature miRNAs.

For other miRNAs, loss of wild type HRPK-1 activity resulted in an increased miRNA abundance, suggesting that HRPK-1 may normally inhibit their processing or reduce their stability (Fig 8B). Here, the changes in miRNA levels were again more significant in the *hrpk-1(tm5522)* mutants than *hrpk-1(zen17)*, correlating with the more severe phenotypes caused by this allele. For miRNAs whose levels change in *hrpk-1* mutants, we did not observe a corresponding change in the levels of their associated miR* strands (S1 Table). While this observation may point to a potential role for HRPK-1 in miRNA stability rather than miRNA processing, miR* strands are known to degrade at a much faster rate than mature miRNAs. As our data captures only a static snapshot into miR and miR* abundances, we cannot at this point conclusively distinguish between the potential HRPK-1 roles in miRNA processing versus miRNA stability.

How might HRPK-1’s role as a positive regulator of miR-84 activity be reconciled with the apparent increase in miR-84 levels in *hrpk-1* mutant animals? It is possible that in addition to the HRPK-1’s function in miRNA processing or stability, it may act to promote the mature complex interaction with the target mRNAs (Fig 8B). Here, a complete loss of HRPK-1 may result in an overall increase of miR-84, but its absence may reduce or inhibit miR-84 activity, perhaps due to the reduced ability of miR-84 to find its targets (Fig 8B). While abnormal HRPK-1(tm5522) protein may fail to inhibit miR-84 processing, it may also trap miR-84 in ineffective complexes, preventing the mature miRNA both from finding its cognate targets and from being degraded (Fig 8B).

Since we did not detect changes in miR-42 and lsy-6 levels at the embryonic stage, it is possible that HRPK-1 synergizes with these miRNAs at the level of miRISC (Fig 8C). Here, HRPK-1 may promote miRISC activity by enhancing the association between miRISC and target mRNAs or could stimulate the interaction between miRISC and downstream effector complexes (Fig 8C). These interactions could be negatively affected by the antimorphic form of HRPK-1 in *hrpk-1(tm5522)* animals. This model is supported by the observation that loss of HRPK-1 is not required for ALG-1/AIN-1 miRISC assembly, as *hrpk-1(zen17)* animals have an intact ALG-1/AIN-1 miRISC (Fig 7E). Future identification of HRPK-1 RNA binding sites and their proximity to miRNA target sites will help illuminate the model that best describes HRPK-1 activity and will help characterize the physical relationship between the two.

It should be noted that the proposed models are not mutually exclusive. Given the diversity of hnRNP and KH domain proteins’ functions, we must allow for the possibility that multiple, miRISC-dependent and independent HRPK-1 activities could synergize with the miRNA-mediated target regulation. For example, an HRPK-1 role in mRNA processing could increase miRNA site availability. Identifying the impacts of HRPK-1 loss on alternative splicing of mRNAs and the resulting changes in miRNA binding site availability can in the future shed light on the plausibility of this model. We look forward to further investigating the mechanisms through which HRPK-1 and miRNAs cooperate to facilitate gene repression.

Overall, our results demonstrate that HRPK-1, a KH domain RNA binding protein, physically and functionally interacts with miRNA-mediated gene repression. Other KH domain RNA binding proteins have been previously shown to biochemically and genetically interact with the miRNA machinery. These include the *C*. *elegans* translational repressor, a Quaking homolog, and a KH domain protein, GLD-1 [28], [41] and VGLN-1 [24]. Additional KH-domain proteins and other hnRNPs have been detected in large-scale mass spectrometry experiments [41], [14], [42]. The molecular mechanisms by which these RNA binding proteins synergize with miRNA pathways to regulate mRNA expression may be diverse and remain largely unknown. Further studies to understand the mechanism of functional interactions between RBPs, including HRPK-1, and miRNA-mediated gene repression will shed light on how these two classes of post-transcriptional gene regulators cooperate to control animal development.

## Materials and methods

### Strain maintenance, RNAi, and phenotypic assessments

*C*. *elegans* strains were grown in standard conditions on NGM plates using OP50 as a food source [43]. Strains were maintained at 20°C unless otherwise noted. RNAi gene knockdown was performed as previously described [44]. Briefly, PS3662 or OH7310 animals were placed on *hrpk-1* and control RNAi food and the F1 progeny were scored for the *cog-1*::*gfp* presence in vulval and uterine cells. *let-7(n2853)* animals were placed on *hrpk-1*, *dcr-1*, and control RNAi food as embryos, reared at 15°C, and scored for vulval rupture as day 1 adults.

For the *mir-48 mir-241(nDf51)* assay, young adults were scored for seam and hypodermal *col-19*::*gfp* expression, alae formation, and seam cell number. Seam cells numbers were obtained by counting the seam cells located between the pharynx and the anus of a given animal. Phenotypes were scored on either the Zeiss Axioplan 2 or the Leica DM6 upright microscopes equipped with DIC and epifluorescence. For *mir-35-41(nDf50)* assay, animals were scored for sterility, brood size and embryonic lethality. Animal sterility, embryonic lethality, and brood size were assessed by scoring entire broods of individual animals. Animals were considered sterile if they were unable to produce any progeny, dead or alive. Brood size refers to the total number of progeny produced, including dead embryos. Percent embryonic lethality was calculated as follows: # of dead embryos/total progeny (live progeny + dead embryos). Vulval bursting was visually assessed beginning at the Day 1 young adult stage and daily thereafter.

### CRISPR-based genome editing

*hrpk-1(zen15)* and *hrpk-1(zen-17)* deletion alleles were produced using CRISPR/Cas9 genome editing using *hrpk-1* site specific guides 1 and 9, corresponding to the 5’ and 3’ ends of the gene, respectively. The following *hrpk-1* guides were used to generate the deletions: *hrpk-1* guide 1, ACTGTCTGTTCCATTAATAG and *hrpk-1* guide 9, CAAGGCCGTGAACGATTCGG. *hrpk-1(zen17)* was outcrossed seven times and *hrpk-1(zen15)* was outcrossed twice. The entire *hrpk-1* locus was sequenced to confirm the nature of the deletion in each mutant.

Endogenously tagged *hrpk-1*::*gfp* strains were generated by inserting a worm codon-optimized GFP coding sequence and a self-excising cassette (SEC) into the C-terminus of endogenous *hrpk-1* locus just before the stop codon through CRISPR/Cas-9 triggered homologous recombination [45]. The SEC was then excised as described [45]. The donor sequence was generated by subcloning 596 bp upstream of the *hrpk-1* stop codon and 600 bp downstream of *hrpk-1* stop codon into the pDD282 vector [45] using Hi Fi assembly kit (NEB). The *hrpk-1* stop codon was eliminated from the donor sequence to allow in frame GFP tag addition. A PAM site mutation corresponding to guide 9 was included in the donor DNA sequence resulting in the sequence change of [CAAGGCCGTGAACGATTCGGTGG] change to [CAAGGCCGTGAACGATTCGGTCG] immediately upstream of the GFP tag sequence. Animal microinjections were performed as previously described [46]. Six independent *hrpk-1*::*gfp* lines were obtained and the resulting endogenously tagged *hrpk-1*::*gfp* loci was sequenced. Since all six lines looked superficially wild type and showed the same *hrpk-1*::*gfp* expression pattern, a single line was chosen for an in-depth analysis. The *hrpk-1*::*gfp(zen64)* strain was outcrossed twice and assessed for *hrpk-1* functional integrity. *hrpk-1*::*gfp* transgenic line had wild type fertility and embryonic viability (S4 Fig), confirming that the GFP-tagged HRPK-1 retained its wild type activity.

The following primers were used in generation of the *hrpk-1* donor sequence in order to place the GFP sequence at the C-terminus of *hrpk-1*: 5’ homology arm forward primer (5’-CGACGGCCAGTCGCCGGCAGATCGTATGGAGGAGCTATTAC-3’), 5’ homology arm reverse primer (5’-CCTGAGGCTCCCGATGCTCCGACAGATGCACCGAATCGTTC-3’), 3’ homology arm forward primer (5’-GGATGACGATGACAAGAGATAAGTTCTGGTGTTCGTACTCTTC-3’), and 3’ homology arm reverse primer (5’-GACCATGTTATCGATTTCCGCCCTTAGGAGATAATTTGC-3’).

### Extract preparation and immunoprecipitation (IP)

Worm extracts were prepared as previously described [47] with the following modifications. Mixed stage animals were homogenized using a Bullet blender (MIDSCI). Briefly, 300μL worm pellets were mixed with RNAse free Rhino beads (MIDSCI) and 300μL lysis buffer and homogenized at the highest setting for 4 minutes. Homogenate was then moved to a fresh tube and spun at 13,000xg for 20 minutes to clarify the extract. The extracts were then used for immunoprecipitation experiments or were flash frozen in liquid nitrogen and stored at -80°C.

ALG-1 immunoprecipitation and Western blotting were performed as previously described [48] using Protein A Dynabeads (ThermoFisher). HRPK-1 detection was done using a custom rabbit anti-HRPK-1 antibody (Pocono) generated against the C-terminal peptide of HRPK-1 (CVRNSTQGRERFGGSV) at the 1:1000 dilution. HRPK-1 immunoprecipitation was performed using the same method as ALG-1 immunoprecipitation [48], with the anti-HRPK-1 antibody covalently crosslinked to the Protein A Dynabeads (ThermoFisher) using dimethylpimelimidate1. Mouse anti-tubulin antibody (Sigma-Aldrich) was used to detect tubulin as a loading control.

### RNA preparation, Northern blotting, and small RNA sequencing

RNA from mixed stage and staged animals was prepared as previously described [47] with the following modifications. Animal pellets were resuspended in water up to 250μL final volume and mixed with 1ml of Trizol (Fisher Scientific) and RNAse free Rhino beads (MIDSCI) and homogenized using a Bullet blender (MIDSCI) at the highest setting for 4 minutes. Homogenate was then moved into a fresh tube, mixed with 212μL of chloroform, and spun down to separate the phases. All remaining steps of the RNA purification were performed as previously described [47]. Northern blotting was performed as described [49], using the following probe sequences: mir-84 (TCTACAATATTACATACTACCTCA), mir-58 (ATTGCCGTACTGAACGATCTCA), let-7 (AACTATACAACCTACTACCTCA), and U6 (TTGCGTGTCATCCTTGCGCAGG). Fluorescent signal intensity was measured using ImageJ.

For small RNA libraries preparation, small RNAs were first size selected by gel purification as described in [50]. The size selected RNA was used to construct small RNA libraries using the NEXTflex Small RNA Library Prep kit v3 (Bioo Scientific) and sequenced on the Illumina NextSeq instrument at the Kansas State University Genomic Core. Data analysis was performed as previously described [14]. The total mapped reads across replicates were as follows: N2 embryos, three replicates (total miR reads = 20,295,289), *hrpk-1(zen17)* embryos, three replicates (total miR reads = 27,354,097), N2 L4 larvae, three replicates (total reads = 49,715,720), *hrpk-1(zen17)* L4 larvae, three replicates (total reads = 48,321,775) and *hrpk-1(tm5522)*, two replicates (total reads = 24,710,895). Differential expression analysis was subsequently performed using the DEseq package [51]. Sequence data files are available in the GEO database under the accession number GSE137831.

### Microscopy and statistics

Localization of the endogenously tagged HRPK-1::GFP transgene was imaged using Zeiss Axioplan 2 upright microscope equipped with a Zeiss Axiocam HR digital camera. Images were assembled using Photoshop. Contrast and brightness of images were not adjusted. F-test was used to analyze brood size, t-test was used to analyze embryonic lethality, and chi-square was used to analyze all other phenotypic data.

### Strains used in this study

The following strains were used in this study: N2 *(wild type)*, UY44 *(hrpk-1(zen15))*, UY38 *(hrpk-1(zen17))*, UY39 *(hrpk-1(zen17); maIs105[col-19*::*gfp]*, UY42 *(hrpk-1(tm5522))*, UY43 *(hrpk-1(tm5522); maIs105[col-19*::*gfp])*, OH3646 *(otls114[Plim-6*::*gfp + rol-6(su1006)]; lsy-6(ot150))*, UY46 *(hrpk-1(zen17) otIs114[Plim-6*::*gfp + rol-6(su1006)]; lsy-6(ot150))*, UY54 *(hrpk-1(zen17) otIs114[Plim-6*::*gfp + rol-6(su1006)])*, UY9 *(hrpk-1(tm5522) otIs114[Plim-6*::*gfp + rol-6(su1006)]; lsy-6 (ot150))*, UY18 *(hrpk-1(tm5522) otls114[Plim-6*::*gfp + rol-6(su1006)])*, PS3662 *(syIs63[cog-1*::*gfp + unc-119(+)])*, OH7310 *(otIs193 [cog-1p*::*lsy-6hp + rol-6(su1006)] syIS63[cog-1*::*gfp + unc-119(+)])*, MT14119 *(mir-35-41(nDf50))*, UY56 *(hrpk-1(zen17)/ht2gfp; mir-35-41(nDf50))*, UY55 *(hrpk-1(tm5522)/ht2gfp; mir-35-41(nDf50))*, VT1296 *(mir-241*,*mir-48(nDf51) maIs105[col19*::*GFP])*, UY67 *(hrpk-1(zen17); mir-241 mir-48(nDf51) maIs105[col19*::*gfp])*, UY68 *(hrpk-1(tm5522); mir-241 mir-48(nDf51) maIs105[col19*::*gfp])*, HML11 *(maIs105[col19*::*gfp] lin-2 (e1309) let-7 (n2853))*, UY157 *(hrpk-1(zen17); maIs105[col19*::*gfp] lin-2 (e1309) let-7 (n2853))*, VT3841 *(alg-1(tm492))*, UY66 *(hrpk-1*::*gfp(zen64))*, MT7626 *(let-7(n2853))*, and UY238 *(maIS105[col-19*::*gfp]; lin-2(e1309))*.

## Supporting information

S1 Table miRNA abundances in hrpk-1 mutants compared to wild type. (XLSX)

S2 Table DEseq analysis of miRNA abundances in hrpk-1(zen17) vs wild type embryos. (XLSX)

S3 Table DEseq analysis of miRNA abundances in hrpk-1(zen17) vs wild type L4 larvae. (XLSX)

S4 Table DEseq analysis of miRNA abundances in hrpk-1(tm5522) vs wild type L4 larvae. (XLSX)

S1 Fig hrpk-1(zen17) and hrpk-1(zen15) show similar phenotypes. *hrkp-1(zen15)* and *hrpk-1(zen17)* produce similar levels of sterility (**A**), embryonic lethality (**B**), and brood size (**C**). An increase in embryonic lethality in *hrpk-1(zen15)* mutant animals (**D**) is most likely due the difference in the number of outcrosses between the strains, with *hrpk-1(zen17)* being outcrossed seven times, while *hrpk-1(zen15)* was outcrossed only twice. ***p≤0.*001*.
(TIF)

S2 Fig ALG-1 immunoprecipitation does not co-precipitate abundant hnRNP protein and hnRNPA1 homolog, HRP-1. (TIF)

S3 Fig hrpk-1(tm5522) is weakly semi-dominant and hrpk-1 is required maternally. Genetic analyses of *hrpk-1(zen17)* and *hrpk-1(tm5522)* alleles reveal that *hrpk-1* activity has a maternal component for some developmental processes such as fertility (**A**), brood size (**B**), and embryonic viability (**C**), but not for vulval integrity in day 3 or older adults (**D**). *hrpk-1(tm5522)* appears to be weakly semi-dominant as evidenced by the presence of defects observed in *hrpk-1(tm5522)/+* and *hrpk-1(tm5522)/hrpk-1(zen17)* animals (**A-D**). Genotype of the score animals is shown. m- indicates that scored animals came from homozygous mutant mothers, m+ indicates that scored animals were progeny of wild type mothers. *hrpk-1(tm5522)m/hrpk-1(zen17)p* animals came from a cross between *hrpk-1(tm5522)* mothers and *hrpk-1(zen17)* fathers. *hrpk-1(tm5522)p/hrpk-1(zen17)m* animals came from *hrpk-1(zen17)* mothers and *hrpk-1(tm5522)* fathers.
(TIF)

S4 Fig GFP-tagged endogenous HRPK-1 retains its activity. C-terminal HRPK-1 GFP tag does not affect animal fertility (**A**) or embryonic viability (**B**).
(TIF)
