# Supplementary figures and images for: HRPK-1, a conserved KH-domain protein, modulates microRNA activity during Caenorhabditis elegans development
Source: PLoS Genet. 2019 Oct 4;15(10):e1008067. doi: 10.1371/journal.pgen.1008067 (PMC6795461; doi:10.1371/journal.pgen.1008067)

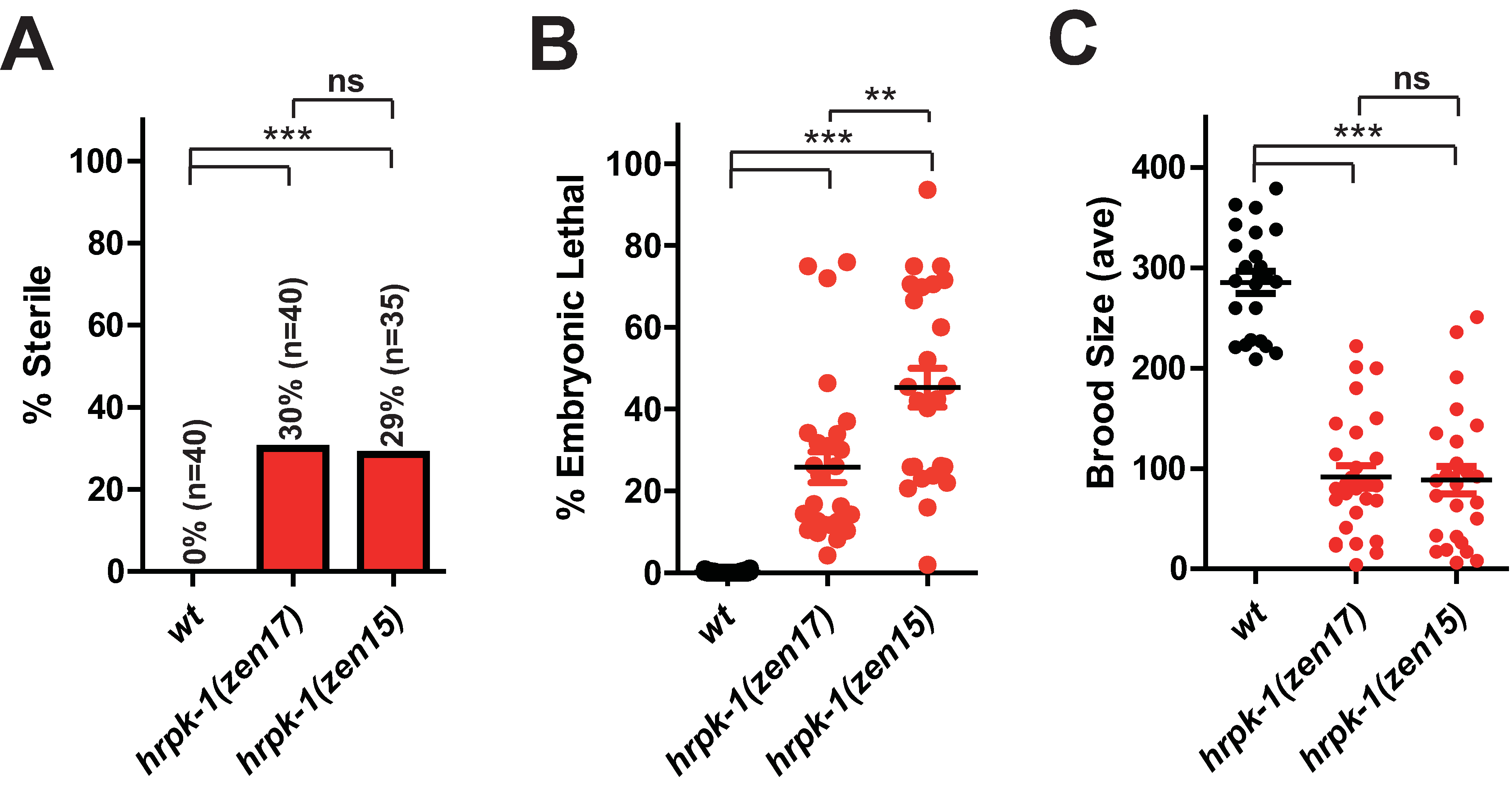

Supplement: S1 Fig — hrkp-1(zen15) and hrpk-1(zen17) produce similar levels of sterility (A), embryonic lethality (B), and brood size (C). An increase in embryonic lethality in hrpk-1(zen15) mutant animals (D) is most likely due the difference in the number of outcrosses between the strains, with hrpk-1(zen17) being outcrossed seven times, while hrpk-1(zen15) was outcrossed only twice. ***p≤0.001. (TIF) [file pgen.1008067.s005.tif]

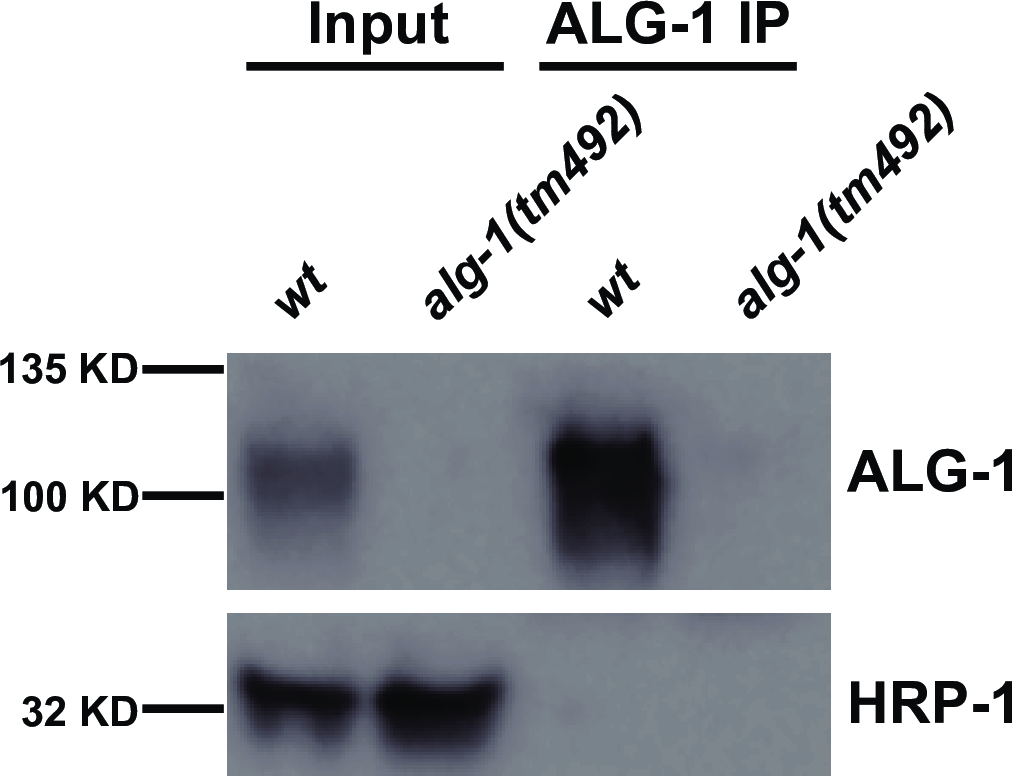

Supplement: S2 Fig — (TIF) [file pgen.1008067.s006.tif]

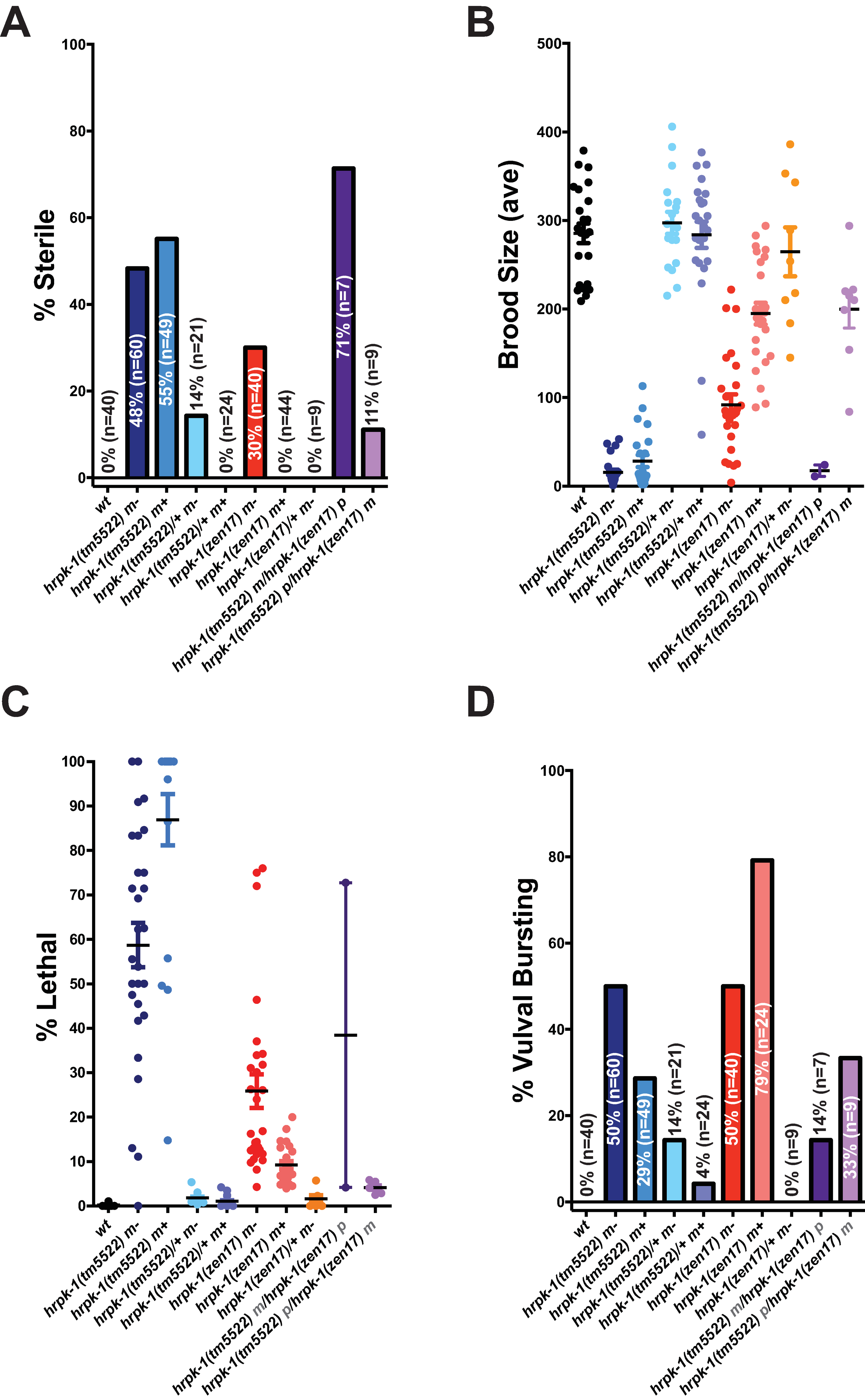

Supplement: S3 Fig — Genetic analyses of hrpk-1(zen17) and hrpk-1(tm5522) alleles reveal that hrpk-1 activity has a maternal component for some developmental processes such as fertility (A), brood size (B), and embryonic viability (C), but not for vulval integrity in day 3 or older adults (D). hrpk-1(tm5522) appears to be weakly semi-dominant as evidenced by the presence of defects observed in hrpk-1(tm5522)/+ and hrpk-1(tm5522)/hrpk-1(zen17) animals (A-D). Genotype of the score animals is shown. m- indicates that scored animals came from homozygous mutant mothers, m+ indicates that scored animals were progeny of wild type mothers. hrpk-1(tm5522)m/hrpk-1(zen17)p animals came from a cross between hrpk-1(tm5522) mothers and hrpk-1(zen17) fathers. hrpk-1(tm5522)p/hrpk-1(zen17)m animals came from hrpk-1(zen17) mothers and hrpk-1(tm5522) fathers. (TIF) [file pgen.1008067.s007.tif]

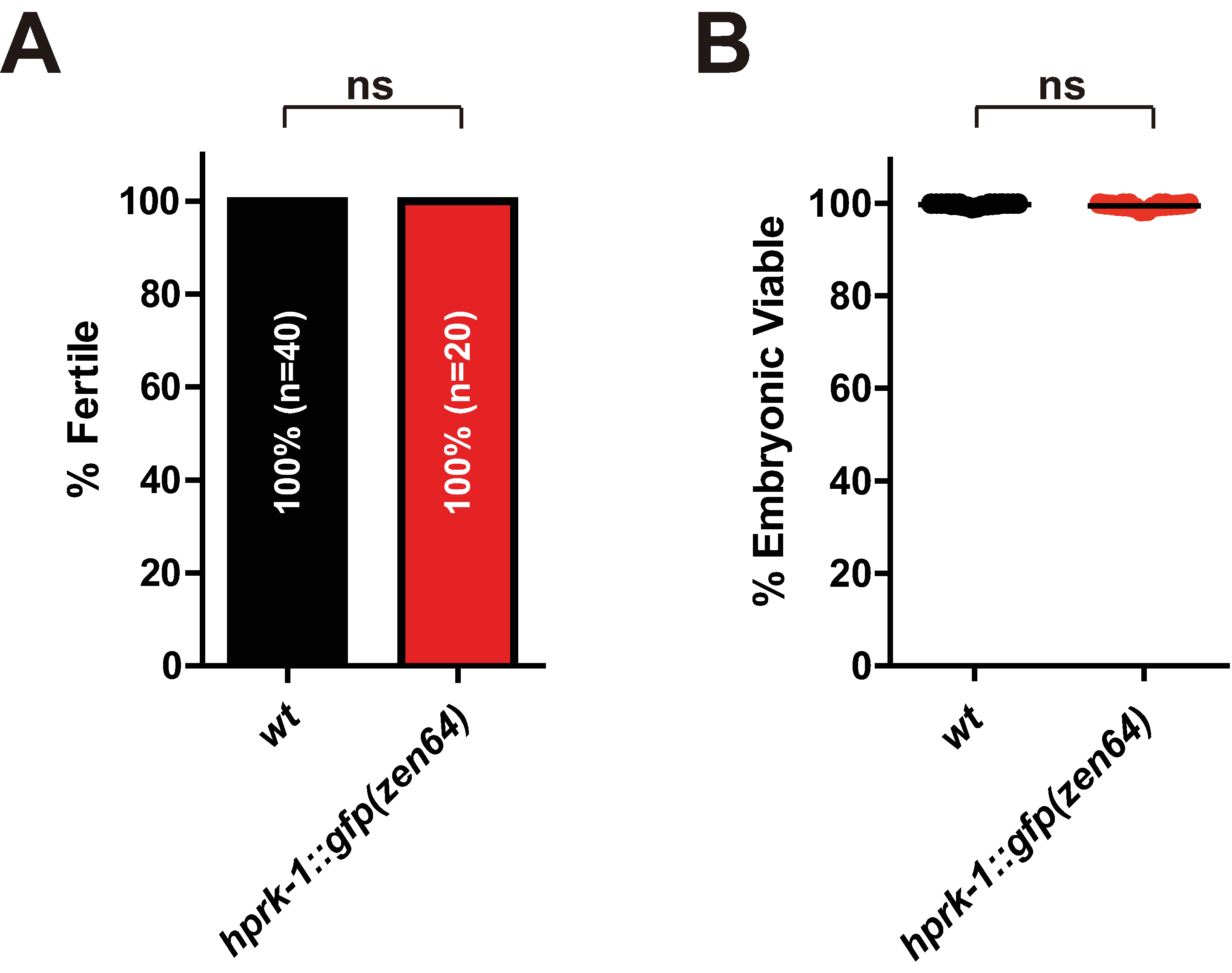

Supplement: S4 Fig — C-terminal HRPK-1 GFP tag does not affect animal fertility (A) or embryonic viability (B). (TIF) [file pgen.1008067.s008.tif]
